# The effect of versican G3 domain on local breast cancer invasiveness and bony metastasis

**DOI:** 10.1186/bcr1751

**Published:** 2007-07-27

**Authors:** Albert JM Yee, Margarete Akens, Bing L Yang, Joel Finkelstein, Peng-Sheng Zheng, Zhaoqiong Deng, Burton Yang

**Affiliations:** 1 Department of Surgery, University of Toronto, Sunnybrook Health Sciences Centre, 2075 Bayview Avenue, Rm MG 371-B, Toronto, Ontario, M4N 3M5, Canada; 2 Sunnybrook Health Sciences Centre, 2075 Bayview Avenue, Rm S-110, Toronto, Ontario, M4N 3M5, Canada

## Abstract

**Introduction:**

Increased versican expression has been associated with local breast cancer invasiveness and a more aggressive tumor phenotype. The cellular mechanisms are not fully understood and this study evaluated versican G3 domain with its EGF-like motifs in influencing tumor invasion and metastasis.

**Methods:**

One recombinant construct was synthesized (a signal peptide for product secretion and the versican G3 domain). The construct was stably transfected into human breast carcinoma MT-1 cells. Cell viability *in vitro *was evaluated in low serum and serum starvation conditions. *In vivo *study of tumor growth was evaluated in a nude mouse model. G3 effects on rodent vascular endothelial cells were evaluated *in vitro *on cell survival, apoptosis, migration, and vascular formation. The effects of VEGF, fibronectin, and G3 on vascular formation were examined. An intracardiac injection model of metastatic human breast carcinoma tested the effect of G3 on distant bony and soft tissue metastasis. Analysis of metastatic burden included histology, radiographs, and micro-CT quantification of osteolysis.

**Results:**

A greater viability of cancer cells was observed in low serum and serum-free conditions in the presence of versican G3. Larger subcutaneous tumors were obtained in the G3 group following tumor cell injection into CD1 mice. G3 induced a greater degree of rodent vascular endothelial cell proliferation and migration *in vitro*. Simultaneous presence of fibronectin, VEGF, and G3 promoted endothelial cell migration in wound-healing assays as compared to the treatments containing none, one or two of these molecules. Systemic tumor burden to distant bony and soft tissue metastatic sites was greater in the G3 group using the intracardiac injection metastatic model

**Conclusion:**

Versican G3 domain appears to be important in local and systemic tumor invasiveness of human breast cancer. Effects include enhancing cell viability, proliferation, migration and enhancing local tumor growth. Potential effects on angiogenesis include enhancing vascular endothelial proliferation, migration, and vessel formation. The interactions between tumor cells, surrounding stromal components and neo-vascularization in breast cancer may include interactions with VEGF and fibronectin. The propensity of versican G3 to influence tumor invasion to bone and the mechanisms of G3 mediated osteolysis warrants ongoing studies.

## Introduction

Versican is a large extracellular proteoglycan that is expressed in a variety of tissues. It was originally isolated from human fibroblasts and developing chicken limb buds during prechondrogenesis when primary mesenchymal cells differentiate into chondrocytes [1-4]. This complex process involves cell division, adhesion, migration, differentiation and production of extracellular matrix (ECM) molecules. Similar to other members of this large aggregating proteoglycan, common features include the presence of N-terminal G1 and C-terminal G3 domains, and a large chondroitin sulfate side chain-bearing sequence localized in the middle region. The G1 domain of versican binds hyaluronan and the G3 domain consists of a lectin-like carbohydrate recognition domain (CRD), and epidermal growth factor (EGF)-like and complement-binding protein-like subdomains. Like other chondroitin sulfate proteoglycans, versican has been reported to inhibit the adhesion of cells to substrata [5]. Versican's activity on cell adhesion varies and both anti-adhesive and adhesive properties have been reported [5-12]. Several studies support the ability of versican to function as an anti-adhesive molecule with activity that may reside in the G1 region [5-8]. Versican may also repress focal contact formation and inhibit cell adhesion [7]. Versican has been reported to interfere with the attachment of cells to various extracellular matrix components such as collagen I, fibronectin, and laminin [13]. It also appears that versican can inhibit intercellular adhesion of normal as well as malignant tumor cells [5,8,14]. The G3 domain of versican has been observed to interact with β1 integrin in glioma cells activating FAK and promoting cell adhesion [9]. Versican also binds to adhesion molecules including L and P selectin on the surface of inflammatory leukocytes [10-12]. Alternate splicing and different breakdown products of versican may in part explain the molecules varying biologic activities in different tissues.

Immunolocalization of versican in breast tumors, including infiltrating ductal carcinoma, has been reported [15]. Of interest is the observation that peripheral areas of infiltrating ductal carcinoma have intense versican expression [15]. This suggests that versican, a molecule with properties that influence cell adhesion, may play an important role in tumor invasion [6,8,14-16]. Extracellular PG-M/versican has been observed to be elevated in a variety of human tumors including breast carcinoma [15,17-21]. As a recognized modulator of cell adhesion and motility for mesenchymal cells, increased versican expression in malignant derivatives appears to contribute towards a more aggressive phenotype [20,21]. Accumulation of versican in breast and prostate tumors appears to be a negative predictor of survival [21,22].

The importance of versican G3 with its EGF-like motifs on local tumor invasion has been demonstrated in other cancer cell types [23-25]. The ability of a local tumor to grow beyond a critical size appears to relate to the formation of vascular stroma, the involvement of a variety of cell types including malignant epithelial cells, surrounding stromal cells, and vascular endothelium. The interaction between tumor cells, stromal components, and growth factors that regulate cell division, adhesion, migration and differential gene expression contribute to this growth. In cell culture versican is expressed only when cells are actively proliferating. Once cells reach confluence, versican expression decreases [26]. Versican is also highly expressed in some types of tumors where cells are actively undergoing proliferation [15,17,18]. Versican's effect on proliferation may be related to its C-terminal G3 domain [6,16,27]. In astrocytoma, mechanisms appear to include versican G3 interactions with β1 integrin and angiogenic factor VEGF [24,25].

Given the knowledge of systemic metastasis in breast cancer to preferentially seed certain anatomic sites (i.e. bone), the mechanisms of breast cancer invasiveness and metastasis in particular to bone is also of interest. The potential role of anti-adhesive molecules such as versican in systemic tumor invasiveness of breast carcinoma has not been extensively evaluated. Versican is highly expressed during the development of long bones in rats up to 6 weeks post partum. Immunoreactivity appears intense at the stage of woven bone and weaker in lamellar bone [28]. Versican expression may be important during the process of tumor bony invasion and subsequent remodeling of bone that leads to osteolysis. We hypothesize that the G3 domain of versican influences not only the local tumor invasiveness in breast cancer but also systemic invasiveness of metastatic breast carcinoma to bone and soft tissues. The present study evaluated this hypothesis *in vitro *and *in vivo *in human MT-1 breast cancer cells.

## Materials and methods

### *In vitro *tumor cell viability

One recombinant construct was synthesized as described previously [27]. In brief, the cDNAs of chicken versican G3 domain was subcloned in a mammalian expression vector (pcDNA3). Shinomura *et al*. determined the entire cDNA sequence of the core protein of chicken PG-M and the deduced sequence revealed a high homology to the corresponding domains of human versican [2]. A leading peptide of link protein (nucleotides 1–180) was joined with versican G3 to facilitate secretion of the gene products. This leading peptide possesses an epitope recognized by the monoclonal antibody 4B6. In addition, a His epitope was added to the C-terminus of the constructs for staining and purification purposes. The G3 construct and a control vector were stably transfected into human breast carcinoma MT-1 cells using Lipofectamine 2000. The MT-1 cells used in the study were provided by Dr O Engenbraaten (Norwegian Radium Hospital, Oslo, Norway) [29]. Recombinant proteins containing C-terminal His-tag in the culture media were subjected to purification using Ni-NTA affinity columns under native directions. The purity of G3 peptides was confirmed by analysis on SDS-PAGE and western blots probed with monoclonal antibody 4B6 recognizing an epitope on the leading peptide.

Human MT-1 cells stably transfected with the G3 construct or the control vector were cultured at a density of 1 × 10^5 ^cells/ml/well on 12-well tissue culture plates in RPMI-1640 media (Life Technologies, Inc. Rockville, Maryland) supplemented with 10% fetal bovine serum (Seromed Inc., Berlin, Germany). Cells were cultured at 37°C in a humidified incubator containing 5% CO_2 _for two days. On day 3, culture media was changed to either serum-free media or media containing 1% fetal bovine serum. Cell numbers were counted on days 4, 5, 6, and 7. The experiment was repeated in triplicate for each tumor cell group and culture media was evaluated for consistent construct expression. Statistical analysis was performed by non-parametric tests for two independent samples comparing cell viability at the time-points indicated previously. Statistical significance was set at p < 0.05.

### *In vivo *G3 effects on local tumor growth

A rodent model of human MT-1 cells inoculated into 6-week old CD1 strain nude mice was used. Following appropriate institutional animal care committee approval, 5 × 10^6 ^cells were inoculated subcutaneously into the dorsal paraspinal tissues. Animals were randomized to either human MT-1 cells containing either versican G3 domain (G3) or the control vector. Human breast cancer cells MT-1 were cultured in RPMI media at 37°C with 5% CO_2_. At 70% to 80% subconfluency, the cells were given fresh media 24 h before inoculation into the mice. Cell viability was determined by trypan blue exclusion, and cells were suspended with greater than 95% viability without cell clumping. We then injected 5 × 10^6 ^cells in 0.2 mL serum-free RPMI media into the dorsal subcutaneous paraspinal region of each rat using a 1 mL syringe with a 26 G needle. Animals were recovered from anesthesia and permitted ad lib cage activity. Tumors were measured weekly thereafter. Tumor volume (V) was estimated using a caliper by measuring the maximal length (L) and width (W), where V = (L × W^2^)/2. Statistical analysis was performed using non-parametric tests for two independent samples with statistical significance set at p < 0.05.

Six weeks after injection, animals were killed by CO_2 _inhalation for further analysis. At necroscopy, tumors were excised, lysed and proteins analyzed by Western blot probed with monoclonal antibody 4B6 that recognizes an epitope at the N-terminal G3 domain [14]. In brief, the lysates were sonicated and cleared by centrifugation. The supernatant was subjected to SDS-PAGE and electroblotted onto a nitrocellulose membrane (Bio-Rad) in 1 × TG buffer (Amresco) containing 20% methanol. The membrane was blocked in TBST (10 mM Tris-Cl, pH 8.0, 150 mM NaCl, 0.05% Tween 20) containing 10% non-fat dry milk powder (TBSTM) for 1 h at room temperature, and then incubated with primary antibodies at 4°C overnight. The membranes were washed with TBST (3 × 30 min) and then incubated with appropriate horseradish peroxidase-conjugated secondary antibodies in TBSTM for 1 h. After washing as above, the bound antibodies were visualized with an ECL kit according to the manufacturer's instructions (Amersham).

### *In vitro *effects of G3 on endothelial cells

For these experiments, rat endothelial cells (cell line Ypen-1; CRL-222 immortalized by Adenovirus-12 SV40 hybrid virus; American Type Culture Collection, Rockville, Maryland) were used. For the cell proliferation assay, endothelial cells were seeded to 12-well tissue culture plates at a density of 1 × 10^4 ^cells/well in IMDM containing 5% FBS and maintained at 37°C overnight. After 12–16 h, culture medium was removed and the cultures were washed with PBS, followed by addition of 1% FBS/IMDM that had been pre-incubated (48 h) with G3 or vector transfected cells. Three days after medium change, endothelial cells were harvested and the cell number was determined with a Coulter counter.

For the cell migration (wound-healing) assay, endothelial cells were seeded to six-well tissue culture plates at a density of 3 × 10^5 ^cells/well in IMDM containing 5% FBS and the cultures were maintained until they reached 95% confluence. The cultures were wounded by scoring with sterile micropipette tips, then washed and fed with 1% FBS/IMDM, which had been pre-incubated (48 h) with G3 or vector transfected cells. After 36 h, the cultures were photographed using a low-magnification microscope. As well, the wounded cultures were incubated with medium containing VEGF and fibronectin at the desired concentrations, followed by photography.

For the cell survival and apoptosis assay, endothelial cells (2 × 10^5 ^cells) were seeded on six-well tissue culture plates to obtain monolayer cultures, and culture medium was replaced with 1% FBS/IMDM, which had been pre-incubated (5 days) with vector or G3 transfected cells. The cultures were maintained at 37°C for 36–48 h. Detached cells were removed and the cultures were washed. The cultures were examined and photographed under a light microscope. Adherent cells were harvested with trypsin/EDTA and the cell number was counted.

After medium change, the cultures were also maintained for 24 h. Cell morphology was examined under a light microscope. Cells adhered to the plates and detached cells in the medium were harvested and combined. Total DNA was prepared, which included those released to the cytoplasm due to fragmentation and those staying in the nucleus, and analyzed on agarose gel to detect DNA fragmentation using a previously reported technique [30].

To evaluate the formation of vessel-like structures, endothelial cells (2 × 10^5 ^cells) were inoculated on six-well tissue culture plates to obtain monolayer cultures, and culture medium was replaced with IMDM that had been pre-incubated (48 h) with vector- or G3 transfected cells and that contained 0.5% or 1% FBS. The cultures were maintained at 37°C for 3–4 days to assess the effects of G3 containing medium.

### *In vivo *G3 effects on metastasis

A rodent intracardiac injection metastatic osteolytic model of human breast carcinoma was used [31]. Following institutional animal care and use committee approval, vertebral metastases were generated by the injection of human breast cancer carcinoma cells (MT-1 – G3 or vector control) into nude rats (*rnu/rnu*; Harlan Spraque Dawley, IN, USA). Human MT-1 cells expressing versican G3 or vector control constructs were cultured at a density of 1.2 × 10^6 ^cells/ml in 175 cm^2 ^flasks in RPMI-1640 media (Life Technologies, Inc. Rockville, MD, USA) supplemented with 10% fetal bovine serum (Seromed Inc., Berlin, Germany) and 1 mg/ml Geneticin^® ^(Invitrogen, Burlington, Ontario, Canada). A total of 15 5–7 weeks old female rats were randomly allocated to either the versican G3 experimental group or vector control group. Each animal was injected with 2 × 10^6 ^cells in 200 μl RPMI-1640 media into the left heart ventricle using a 1 ml syringe with a 25 G needle under general anaesthesia (2% isofluorane/2 L O_2_/min). Pulsatile blood within the needle cone confirmed the correct position of the needle. The animals were immediately recovered and returned to their cages with free access to food and water. The animals were clinically assessed daily and their weight was recorded once a week. Rats were killed at 24 days or at acceptable tumour endpoints for animal care. The rats were killed with a barbiturate overdose (120 mg/kg Euthanol^®^, Bimeda-MTC, Cambridge, Canada).

For analysis, fine detail radiographs were taken (Model MX-20; Faxitron X-ray Corp., Wheeling, IL, USA) of murine appendicular bones. Digital images of scapular osteolysis were analyzed using Image-Pro Plus (Media Cybernetics, Silver Spring, MD, USA) and an areal (mm^2^) quantification of osteolysis performed comparing experimental to control groups. Necroscopy was performed and solid organs were evaluated for macroscopic tumors. In addition, histological evaluation of tumour burden was performed on bony sites including the humeri, femora, lumbar spine and solid organ sites including the brain, lung, liver, spleen and kidney. The slides were stained with haematoxylin and eosin as well as with an antibody to human EGFR (Zymed EGFr Kit #28-0005; Zymed Laboratories Inc., San Francisco, CA, USA). This antibody does not crossreact with rat tissue thereby facilitating the identification of human cells within murine vertebrae. The lumbar vertebrae (L1–L4) of experimental and control (vector) animals underwent micro-CT scanning for volumetric measurements of tumour burden prior to histology analyses (resolution: 34.7 μm × 34.7 μm × 34.7 μm/voxel). Vertebral bodies of each rat were individually analyzed. The outer vertebral bodies were segmented using a semi-automated level set method. The pedical–vertebral body interface and greater region of interest was manually defined with a flattened cylinder. The exterior of the vertebral body cortex was automatically detected with a level set method. (ITK, Bethesda, MD, USA). Mean intensity, standard deviation of intensity and median intensity within the vertebral body were calculated using Amira software (Amira v. 3.1.1, Mercury Computer Systems, Chelmsford, MA, USA). Within the vertebral body, bone and marrow were distinguished using a threshold of 1000 Hounsfield Units (HU) and volumes were calculated. Stereological quantities were calculated using the described volumetric analysis and triangulated surface data. Histomorphometry was performed using Image Scope (Aperio Technologies Inc., Vista, CA, USA). Statistical analyses were performed using non-parametric analysis for two independent samples and analysis of variance for repeated measurements of animal weight over time. Statistical significance level was set at p < 0.05.

## Results

### G3 expression in breast cancer cells

**Figure 1:**
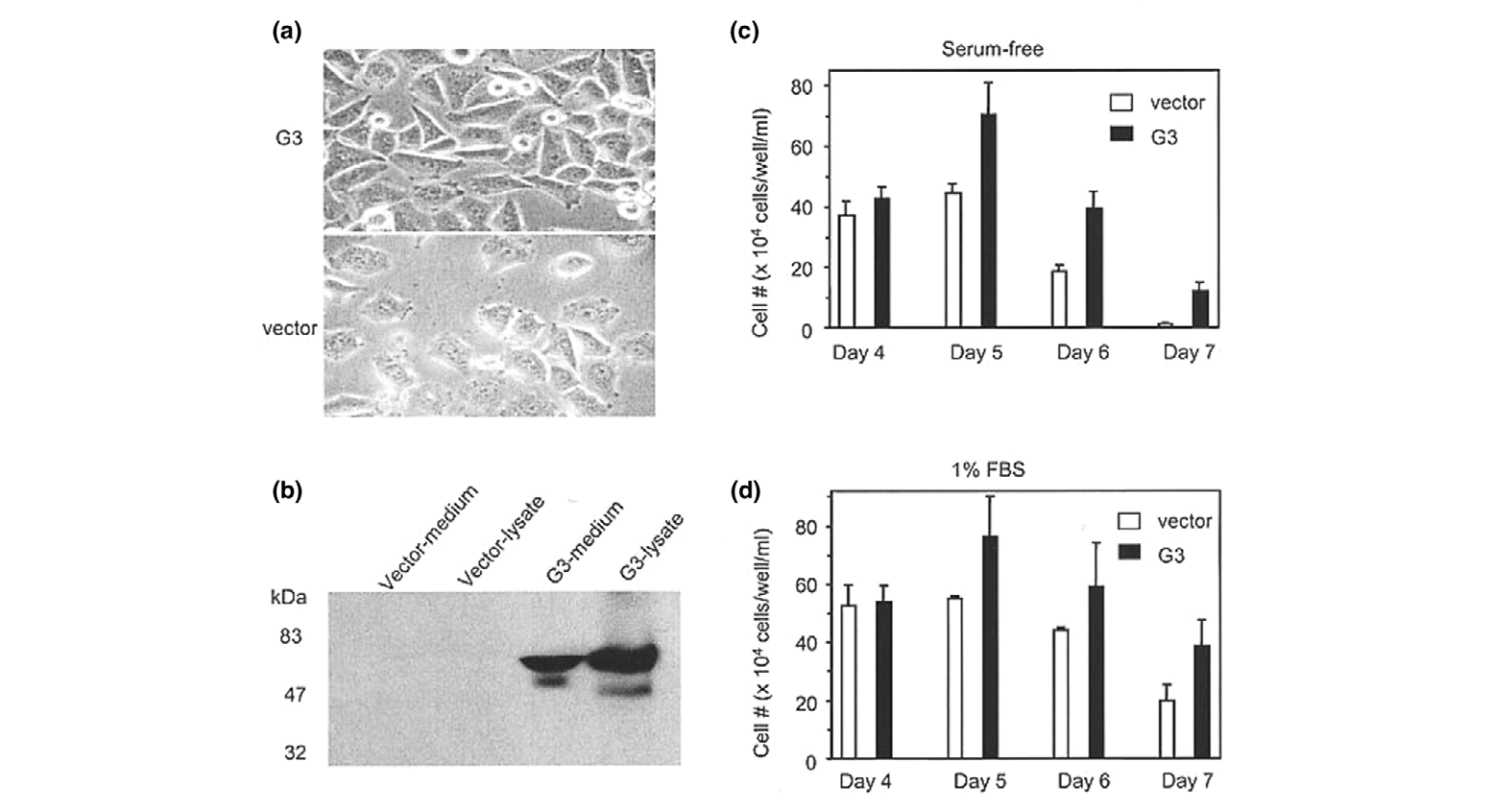
*In vitro *MT-1 cell viability. The morphology of human MT-1 cells *in vitro*, expression of G3 in cell lysate and culture media, and tumor cell viability in serum-free and 1% FBS culture conditions for the G3 experimental group are compared to control. **(a) **Morphologically, cells in the MT-1 G3-transfected MT-1 cells appeared more elongated *in vitro *when compared to the predominant cuboid appearance of cells in the vector control group. **(b) **The expression of G3 in the cell lysate and culture media of MT-1 cells is demonstrated and is contrasted to the vector control group. **(c) **A greater tumor cell viability (mean cell number + SD) was observed in serum-free and **(d) **1% FBS culture conditions for the G3 experimental group as compared with the vector control group (p < 0.05 days 5,6, and 7).

Morphologically, cells in the G3 transfected group appeared more elongated *in vitro *when compared to the predominant cuboid appearance of cells in the vector control group suggesting an enhancement of cell elongation by the G3 product (Figure 1a). The expression of G3 in the cell lysate and culture media of MT-1 cells transfected with the G3 construct containing EGF-like motifs is depicted in Figure 1b and is contrasted to the vector control group. Greater tumor cell viability was observed in serum-free and 1% FBS culture conditions for the G3 experimental group when compared to the vector control group (p < 0.05 days 5, 6, and 7; Figure 1c and 1d).

### G3 mediated tumor growth properties *in vivo*

**Figure 2:**
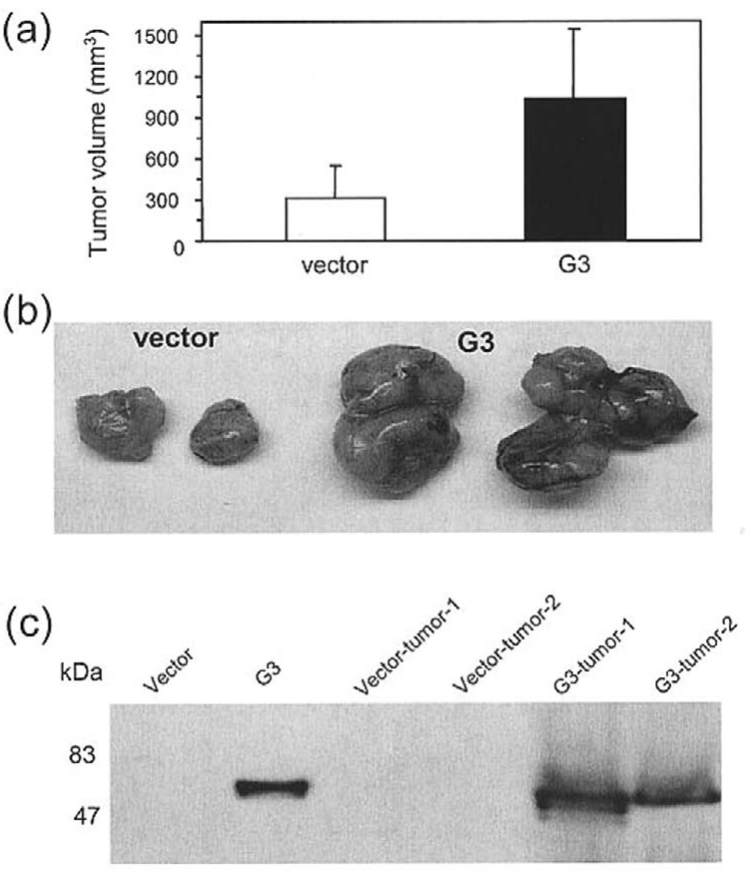
Versican mediated *in vivo *local tumor growth. Greater tumor growth (mean volume mm^3 ^+ SD) was observed in the experimental group of animals inoculated with G3 expressing MT-1 cells. **(a, b) **Animal inoculated with G3-transfected cells grew larger local tumors when compared to the control group (p < 0.05). **(c) **This was consistent with the observation of increased versican G3 expression as assessed by immunoblotting in the experimental group following tumor tissue harvest when compared to the vector control group.

*In vivo*, rodents inoculated subcutaneously with MT-1 cells transfected with the G3 construct containing EGF-like motifs grew larger local tumors when compared to the vector control group (Figure 2a and 2b, mean volume +/- SD in experimental group vs vector controls, p < 0.01). This was consistent with the observation of increased versican G3 expression as assessed by immunoblotting in the experimental group following tumor tissue harvest when compared to the vector control group (Figure 2c).

### G3 mediated effects on endothelial cells

**Figure 3:**
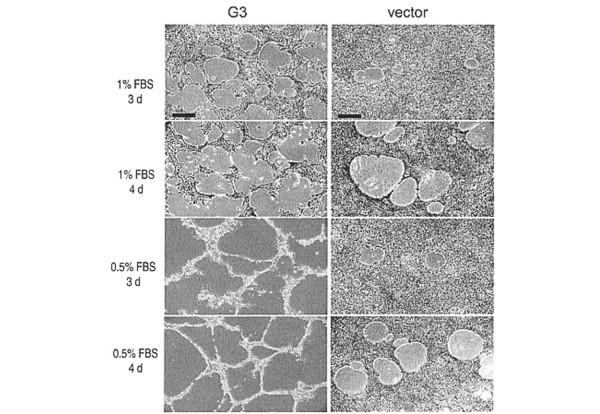
The formation of vessel-like structures in G3-expressing cells. Culture medium in subconfluent endothelial cells was replaced with 0.5% or 1% FBS/culture medium that had been pre-incubated with G3- and vector-transfected cells for 48 h. Three (3 d) or four (4 d) days after medium change, cultures treated with G3-containing medium formed clear vessel-like structures as compared to the vector control. The scale bar depicted represents 250 μm.

**Figure 4:**
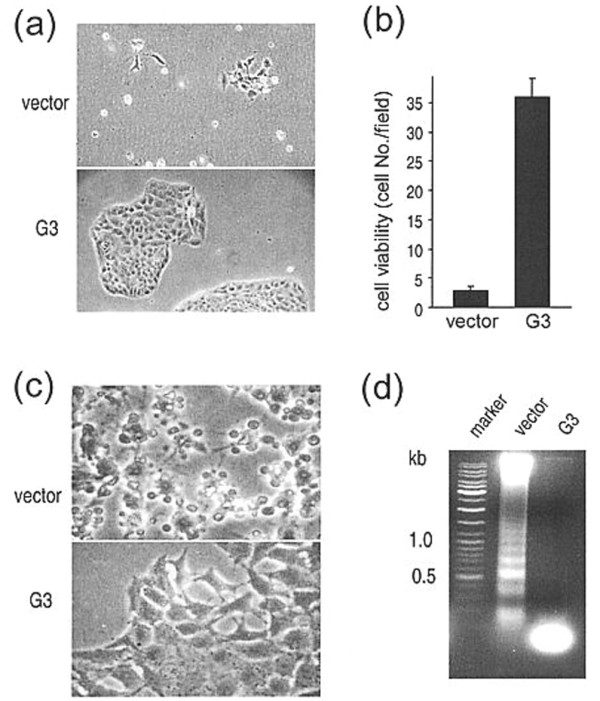
Effect of G3 on endothelial cells. **(a) **G3-containing medium enhances cell attachment and promotes greater cell viability. Culture medium in subconfluent endothelial cell cultures was replaced with 1% FBS/medium that had been preincubated with G3- or vector-transfected cells for 5 days. Two days after medium replacement, spent medium from vector-transfected cells promoted the detachment of cells from plates while spent medium from G3-transfected cells did not. **(b) **After cell detachment, cultures were washed, and the adherent cells were harvested and counted (mean number/field + SD). The difference in cell viability between cultures treated with medium from G3- and vector-transfected cells was significant (p < 0.01). This figure also demonstrates that G3-containing medium enhances cell proliferation and reduces cell apoptosis. **(c) **One day after medium change, cells treated with medium from vector-transfected cells underwent apoptosis, while cells treated with G3-containing medium grew normally. **(d) **DNA was prepared from cytoplasmic and nuclear fractions and subjected to agarose gel electrophoresis. DNA laddering was detected in cells treated with medium from vector-transfected cells.

We studied how G3 affected vessel formation *in vitro *using rat endothelial cells as a culture model. When endothelial cultures were treated with medium obtained from G3 transfected cells, the cultures formed vessel-like structures (Figure 3). The endothelial cultures were treated with 1% FBS-containing medium that had been pre-incubated (5 days) with G3 and vector-transfected cells. G3 containing medium was found to promote cell viability (Figure 4a), and quantitative analysis indicated that the difference between G3 and vector-transfected cells was very significant (Figure 4b; mean number/field ± SD). To examine how these cells died and detached from the plates, cell morphology was monitored carefully. We observed that 24 h after medium change, cells treated with control medium underwent apoptosis, while cells treated with G3 containing medium grew normally (Figure 4c). Evidence of DNA laddering further confirmed apoptosis in the cultures treated with medium from vector-transfected cells (Figure 4d).

### G3 effects on endothelial cell migration with and without the presences of VEGF and fibronectin

**Figure 5:**
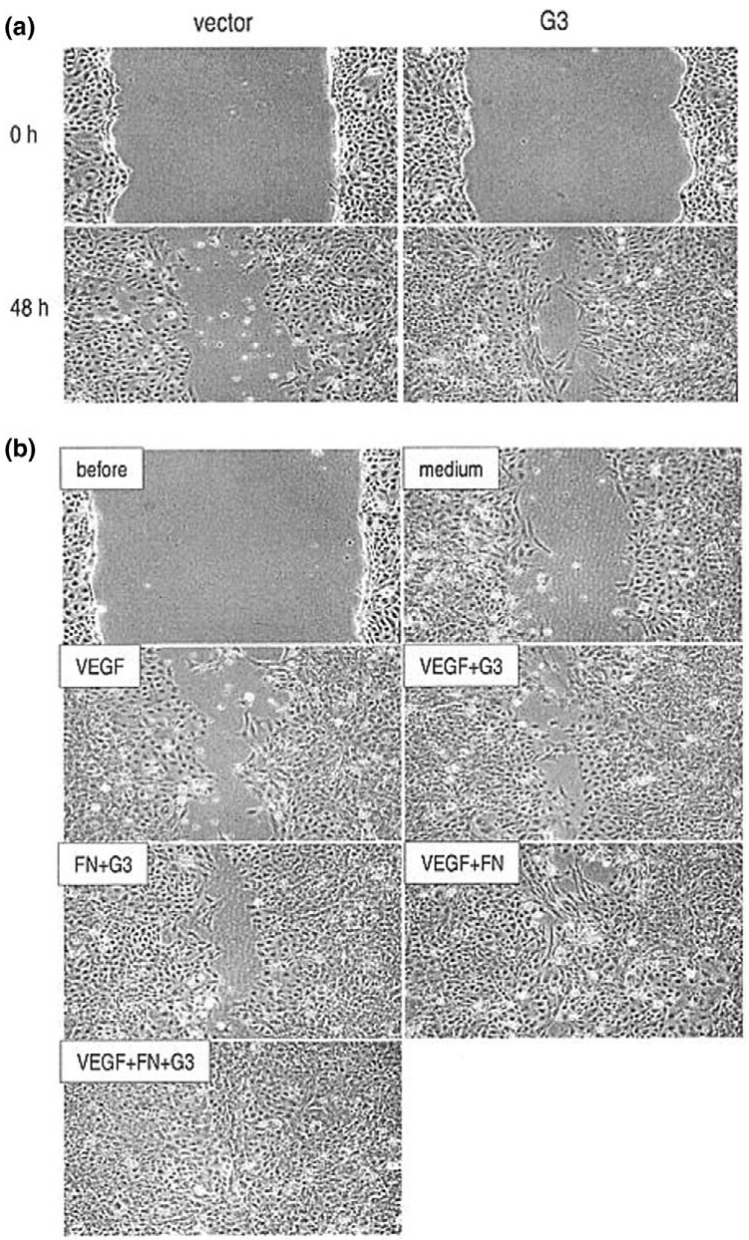
Endothelial cell migration. G3-containing medium enhances endothelial cell migration 48 h following gap formation **(a)**. Monolayer endothelial cell cultures were wounded (0 h) by micro-tips and washed with PBS, followed by addition of culture medium pre-incubated with G3- and vector-transfected cells. Addition of G3-containing medium enhanced endothelial cell migration to the wounding areas. Endothelial cell migration is enhanced in the presence of purified G3, fibronectin (FN) and VEGF **(b)**. Endothelial cells were seeded on 12-well tissue culture plates. The cultures were wounded with micro-tips (before) and washed, and fibronectin, VEGF and purified G3 were added individually or in combination as indicated. In the presence of all three molecules, cells exhibited enhanced migratory capacity. Medium represents culture media in the absence of G3, VEGF, and FN.

In a wound-healing assay, treatment with G3 containing medium greatly enhanced endothelial cell migration to the wounding areas, as compared to control medium (Figure 4e). Our results demonstrated the effects of G3 on endothelial cell activities that are known to be relevant and may contribute to angiogenesis. Combinations of fibronectin, VEGF and purified G3 were tested. In all cases, the simultaneous presence of fibronectin, VEGF and purified G3 promoted endothelial cell migration in wound-healing assays as compared to the treatments containing none, one or two of these molecules (Figure 5).

### *In vivo *G3 effects on systemic metastasis

All rats (15/15) developed metastases in bone and solid organs. Animal weight loss was observed as early as 2–3 weeks following intra-cardiac tumor cell injection. There appeared to be a trend to a more progressive weight loss pattern in the versican G3 group following tumor cell injection, however, the differences were statistically not significant (p = 0.07). At necroscopy, macroscopic tumor burden in soft tissue metastatic sites was greater for the versican G3 group. All animals developed brain and lung metastasis in the versican G3 group when compared to 73% for brain and 27% for lung in the vector control group (p < 0.05). Ovarian and renal tumors were also more prevalent in the G3 group (67%) when compared to 27% of control animals (p < 0.05). The macroscopic size of the observed ovarian metastases in the G3 group was larger when compared to the vector group, a finding that was confirmed by subsequent histological evaluation.

**Figure 6:**
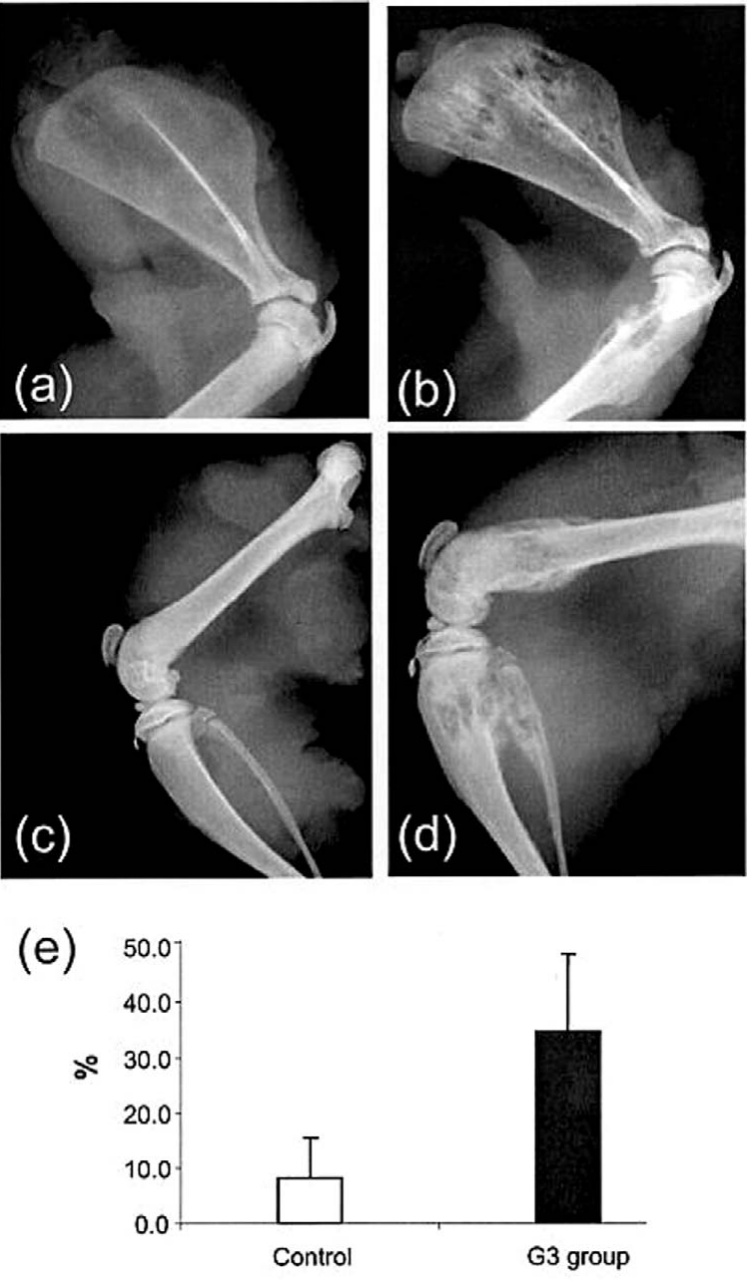
Appendicular osteolysis. Faxitron images of rat shoulder and proximal humerus specimens as well as femura and proximal tibia of vector control rats **(a, c) **and MT-1-G3 rats **(b, d)**. Mean + SD percent osteolysis within the shoulder blade was 8.1 + 8.2 in the control group versus 34.7 + 13.8 in the G3 group **(e)**.

**Figure 7:**
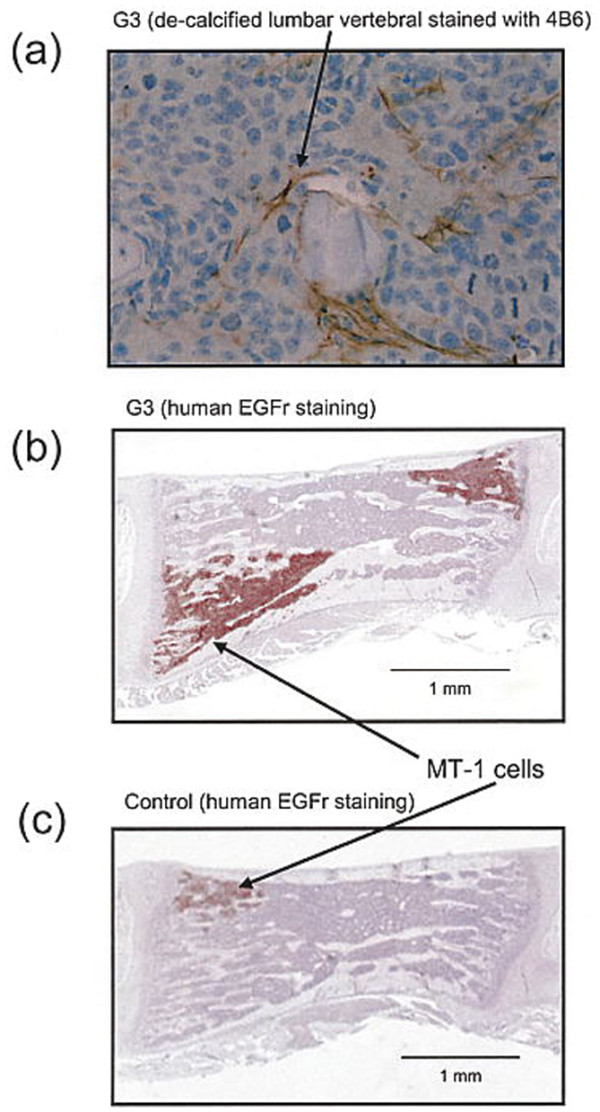
Lumbar vertebral histology. Decalcified lumbar vertebrae section stained with human EGFR antibody and anti-versican G3 domain antibody 4B6 of a rat inoculated with **(a, b) **G3-transfected MT-1 cells when compared to lesser tumor burden observed in a representative control animal **(c)**.

Appendicular osteolysis was observed by 3 weeks using fine detail radiography. The area of osteolysis was significantly higher in the experimental group when compared to the control group (p = 0.002) (Scapular osteolysis shown in figure 6). Vertebral osteolysis as quantified by μCT imaging was significantly greater in the G3 experimental group when compared to the vector-control group. As a result of osteolysis, remaining bone volume/total vertebral volume was 0.69 ± 0.07 (mean ± SD) in the control group and 0.58 ± 0.08 (mean ± SD) in the G3 group; p = 0.003. Concordantly, a greater tumor burden as reflected by histologic staining for hEGFr was observed in harvested lumbar spine of versican G3 rats. The mean ± SD percentage hEGFr staining positivity was 10.0 ± 10.5 control group, 23.7 ± 7.2 G3 group, p = 0.004) (Figure 7). Metastases to bone were confirmed histologically and radiologically in all animals although tumor burden was greater for the G3 group when compared to the vector control group.

## Discussion

The results of the present study indicate that the G3 domain of versican influences breast cancer tumor cell adhesion, migration, proliferation and metastatic tumor burden. A greater viability of human MT-1 breast cancer cells *in vitro *was observed in low-serum or serum-free conditions. This was also consistent with the observations of a greater degree of local tumor growth when evaluated in the *in vivo *rodent model. On average, local tumors were three- to fourfold larger in the G3 experimental animals when compared to vector control animals 6 weeks following subcutaneous tumor cell injection. Consistent with the observation of greater local tumor growth, the G3 domain of versican also influences the development of distal metastasis to bone and soft tissue. Athymic rats injected with versican G3 transfected human breast carcinoma cells (MT-1) were observed to demonstrate a greater systemic tumor burden than animals injected with control cells. Although all animals in the study developed systemic metastasis the number and size of the metastases were larger in the versican G3 group when compared to the vector control group. Bony tumor burden as quantified radiologically was greater for the experimental G3 group.

Extracellular matrices that express high amounts of versican may function as modulators of cell proliferation and migration [32]. Versican expression has been shown to be a predictor of breast cancer survival in women and increased versican expression in the peri-tumoral matrix is correlated with a higher risk of cancer relapse [21]. A significant correlation between tumor grade and degree of versican expression has also been observed in canine colonic adenocarcinomas [33]. The expression of versican by tumor cells themselves appears to vary. Versican expression has been observed in both tumor and stromal cells although the relative extent varies amongst different cancer cell types. The direct expression of versican by tumor cells is variable with a greater expression being associated with highly malignant tumors [34]. The expression of versican by tumor cells appears to relate to higher mitotic activity and a reflection of cell proliferation [34]. Versican mediated cancerous growth appears dependent on the close interactions between tumor cells and its surrounding stromal components. In breast cancer, there does appears to be a greater degree of versican localization in interstitial stromal tissue within tumor cell nests versus that observed in either the tumor cells themselves or in stromal tissue of adjacent non-malignant areas of breast tissue [15,21]. A limitation of the intracardiac injection metastatic model used in the present study is that it bypasses the step of initial establishment of a local tumor with secondary circulatory seeding. A spontaneous mouse mammary model of metastasis may be considered more reflection of the physiologic process of invasion and metastasis however the cells utilized are not of human origin. *In vitro *studies using conditioned media from breast cancer cell lines are able to upregulate versican expression of cancer derived and non-cancer derived mammary fibroblasts without an observed difference in basal level of versican synthesis or the degree of stimulation observed between the two fibroblast groups [21]. The peripheral regions surrounding a tumor appear to have a greater versican expression and the pathways involved in versican-mediated cancerous growth warrants ongoing study.

There is increasing knowledge on the pivotal role of epidermal growth factor receptor (EGFR) together with HER2 in breast carcinoma and how it may influence downstream signal transduction pathways that regulate cell survival and proliferation [35-38]. The presence of two EGF-like motifs in versican G3 given the increasing importance known clinically regarding the role of EGF receptors in invasive breast carcinoma is of interest. Certain breast carcinoma cell lines (e.g. MDA-MB-231) have been reported to over express the receptor for EGF [39]. The breast cancer cell line (MT-1) used in the present study also possess a high cell surface expression of the EGF receptor [29]. Interaction of the EGFR and versican has been demonstrated for transfected astrocytoma U87 cells [25,40]. In addition, versican binds to hyaluronan through its tandem repeats. Versican–hyaluronan complexes increases viscoelasticity around the cells to support cell-shape changes necessary for proliferation and migration [41,42]. It has been shown that hyaluronan production is increased at sites of cell proliferation, tissue remodeling and tumor cell invasion [40].

Neoangiogenesis, the new formation of blood vessels is associated with endothelial cell proliferation, migration, and formation of new capillaries is a response to the increased demand of tumor tissue for oxygen and nutrients. Although no *in vivo *assay for angiogenesis was performed in the present study, we observed *in vitro *that versican G3 influenced vascular endothelial proliferation through mechanisms that include apoptosis. In our study, a greater degree of DNA laddering was observed in the vector control group (Figure 4d) than in the versican G3 experimental group. The migration of rat endothelial cells across a gap *in vitro *also appeared to be greater in the presence of G3 conditioned media. The mechanisms of versican interaction with other cell and extracellular matrix components appear to include cell surface integrins (i.e. β1 integrin), fibronectin, and angiogenic factors (i.e. VEGF) [9,24]. It has been reported that PG-M/versican prepared from chicken fibroblasts and versican-like proteoglycans prepared from human sciatic nerve bind to fibronectin [13,43]. Fibronectin also binds VEGF and regulates endothelial cell activities [44]. We examined if the presence of these molecules affected endothelial cell activities. Combinations of fibronectin, VEGF and purified G3 were tested. In all cases, the simultaneous presence of fibronectin, VEGF and purified G3 promoted endothelial cell migration in wound-healing assays as compared to the treatments containing none, one or two of these molecules (Figure 8).

In this study, metastases developed mainly in tissues where versican is still expressed in human adults and rat tissue [45]. A greater tumor burden was observed in brain, lung, ovary, kidney and bone in G3 rats when compared to vector control. Given the knowledge of systemic metastasis in breast cancer to preferentially seed certain anatomic sites notably bone, the relationship of breast cancer and versican in bone is of interest. Engebraaten *et al*. observed site-specific establishment of metastases dependant on breast cancer cell line evaluated noting that MT-1 cells had a predilection for bony metastasis when compared to solid organ metastases using MA-11 cells [29]. The present study corroborated this site specificity with bony metastasis occurring in all rats although the tumor burden was greater for the G3 experimental group. The role of versican in bone is less well characterized than its expression and function in other soft connective tissues. Nakamura *et al*. evaluated the expression of versican and ADAMTS 1,4,5 (a family of extracellular proteases, which are implicated in cleaving the protein versican) in rat bone development [28]. Versican appears highly expressed during the development of long bones in rats up to 6 weeks post partum. Immunoreactivity appears intense at the stage of immature woven bone and weaker in mature lamellar bone [28]. Versican mRNA was prominent in osteoblasts and corresponded to localization of the protein. The temporal and spatial mRNA expression pattern of ADAMTS 1, 4, and 5 followed that observed for versican. Thus, during bone development woven bone rich in versican alters into lamellar bone containing little versican. Versican expression may also be important during the process of tumor bony invasion and subsequent remodeling of bone that leads to osteolysis with a resultant loss in mature organized bony micro-architecture. The propensity of versican G3 to influence tumor invasion to bone and the mechanisms of versican G3 mediated osteolysis warrants ongoing study. With the known interactions between versican G3 and β1 integrin in other cancer cell types and the increasing knowledge of several β3 integrin-expressing cell populations including osteolasts in breast cancer tumor progression, the known interaction between versican G3 and integrin receptors such as αVβ3 in bone may increase our understanding towards tumor mediated chemotactic and haptotactic migration towards bone factors [25,46].

## Conclusion

In summary, versican is implicated in local tumor invasiveness in human breast carcinoma. The present study suggests a potential significant role of the G3 domain of versican with its EGF-like motif in influencing tumor cell viability, proliferation, and local tumor growth. These effects also include potential effects on endothelial cells that are implicated in neo-angiogenesis. Versican G3 also influences systemic metastasis in a murine model of metastatic human breast carcinoma. The propensity of versican G3 to influence tumor invasion to bone and the mechanisms of versican G3 mediated osteolysis warrants ongoing study.

## Abbreviations

αVβ3 = αVβ3 integrin; β1 = β1 integrin; β3 = β3 integrin; CRD = carbohydrate recognition domain; ECM = extracellular matrix; EGF = epidermal growth factor; EGFR = epidermal growth factor receptor; hEGFR = vascular endothelial growth factor; micro-CT (μCT) = microcomputed tomography.

## Competing interests

The authors declare that they have no competing interests.

## Authors' contributions

The authors' contributions to this research work are reflected in the order shown, with the exception of AJY and MA who contributed equally to the majority of the *in vitro *and *in vivo *experimental work and preparation of the manuscript. BLY, JAF, PZ contributed towards the *in vitro *and *in vivo *work looking at local tumor growth. MH performed the image analysis required for fine detail radiographic and micro-CT quantification of bony tumor osteolysis. ZD contributed towards the *in vivo *mice and rat studies on local tumor growth and metastasis, respectively. BBY and AJY conceived the study and participated in its design and coordination. All authors read and approved the final manuscript.
